# Gut microbial metabolites and the brain–gut axis in Alzheimer’s disease: A review

**DOI:** 10.17305/bb.2025.12921

**Published:** 2025-08-11

**Authors:** Xinchen Ji, Jian Wang, Tianye Lan, Dexi Zhao, Peng Xu

**Affiliations:** 1 College of Traditional Chinese Medicine, Changchun University of Chinese Medicine, Changchun, China; 2 Department of Encephalopathy, The Affiliated Hospital to Changchun University of Chinese Medicine, Changchun, China; 3 Department of Rehabilitation, The Affiliated Hospital to Changchun University of Chinese Medicine, Changchun, China

**Keywords:** Alzheimer’s disease, AD, brain–gut axis, gut microbial metabolites, mechanism

## Abstract

Alzheimer’s disease (AD) is increasingly recognized as a disorder that extends beyond the brain, with accumulating evidence implicating gut microbiota-derived metabolites in its onset and progression. This narrative review synthesises 92 peer-reviewed animal, human and meta-analytic studies published between 2010 and 2025 that investigated short-chain fatty acids (SCFAs), tryptophan-derived indoles and kynurenines, trimethylamine N-oxide (TMAO), and secondary bile acids in the context of AD. Collectively, the literature shows that SCFAs support blood–brain-barrier integrity, dampen microglial reactivity and enhance synaptic plasticity, yet can paradoxically amplify β-amyloid (Aβ) deposition under germ-free or supraphysiological conditions, highlighting the importance of host status and dosing. Beneficial indole metabolites such as indole-3-propionic acid counter oxidative stress, strengthen intestinal and cerebral barriers and suppress pro-inflammatory cascades, whereas a shift toward neurotoxic kynurenines correlates with cognitive decline. TMAO emerges as a consistently deleterious metabolite that aggravates endothelial dysfunction, neuroinflammation and Aβ aggregation; dietary precursor restriction and microbial enzyme inhibitors are therefore being explored as mitigation strategies. Secondary bile acids and polyphenol derivatives further modulate mitochondrial bioenergetics and NF-κB signalling, broadening the therapeutic landscape. Multi-omics profiling reveals that AD patients typically exhibit reduced SCFAs and indoles but elevated TMAO, changes that scale with Mini-Mental State Examination scores, brain atrophy and cerebrospinal Aβ_4__2_ levels. Early probiotic and faecal-microbiota-transplant trials have begun to normalise these metabolite profiles and yield modest cognitive benefits, underscoring translational potential. Altogether, gut-derived metabolites are not passive by-products but active modulators of neural, immune and metabolic circuits along the microbiota–gut–brain axis; their targeted manipulation and standardised metabolomic assessment could enable earlier diagnosis and precision microbiome-based interventions for AD, a promise that now warrants validation in large, longitudinal and mechanistically informed clinical studies.

## Introduction

Alzheimer’s disease (AD) is a progressive neurodegenerative disorder primarily characterized by cognitive decline, memory impairment, language dysfunction, and deficits in executive functioning. The global prevalence of AD is steadily increasing, with projections indicating that the number of affected individuals could exceed 150 million by 2050, placing a substantial burden on public health systems and caregiving resources [1]. Despite advancements in understanding the roles of genetic predisposition, β-amyloid (Aβ) deposition, and tau hyperphosphorylation, the etiology and pathogenesis of AD remain incompletely understood.

Recent research has highlighted the gut microbiota as a potential contributor to the development and progression of AD. As an essential “second genome” of the host, the gut microbiota plays a critical role in nutrient absorption, immune regulation, and metabolic homeostasis. Furthermore, it establishes a bidirectional communication network with the central nervous system through the microbiota–gut–brain (MGB) axis [2, 3]. Accumulating evidence suggests that individuals with AD exhibit reduced gut microbial diversity, lower levels of beneficial microbial metabolites such as short-chain fatty acids (SCFAs), and an increased abundance of pro-inflammatory taxa, including *Proteobacteria* and *Escherichia–Shigella*. This pattern of dysbiosis is closely associated with neuroinflammation and neuronal apoptosis [4, 5].

**Table 1:** Summary of selected literature on gut microbial metabolites in Alzheimer’s disease research

**Researchs**	**Metabolite/ mechanism**	**Study type**	**Model/sample**	**Intervention/ analysis**	**Key findings**	**Year**
Colombo et al. [73]	SCFAs	Animal experiment	Germ-free (GF) vs SPF APPPS1 mice	Oral SCFA supplementation	SCFAs restored Aβ plaque load and microglial activation in GF mice to SPF levels	2021
Bowerman et al. [74]	SCFAs	Animal experiment	APP/PS1 mice	Oral SCFAs supplementation	Altered gut microbiota diversity, but no significant changes in cognition or Aβ pathology	2022
Wasén et al. [75]	B. fragilis (Bacteroidota phylum)	Animal experiment	APP/PS1-21 and 5xFAD mice	B. fragilis administration or clearance	Increased Aβ deposition, inhibited microglial clearance; antibiotic reversal observed	2024
Gao et al. [28]	TMAO	Animal experiment	WT and APP/PS1 mice	Systemic TMAO injection	Promoted synaptic loss, Aβ aggregation, and cognitive impairment	2022
Long et al. [60]	TMAO	Meta-analysis	7 cohort studies	Systematic review and pooled analysis	High TMAO levels significantly associated with cognitive impairment (OR ≈ 1.39)	2024
Zuo et al. [76] (non-primary source)	SCFAs (mechanistic)	Review article	*In vitro* and animal studies	Literature synthesis	SCFAs regulate neuroinflammation via HDAC inhibition, GPCRs, immune modulation	2022

*Note:* 1. Zuo et al. (2022) is a review article and does not present primary experimental data. Included for mechanistic reference. 2. Wasén et al. reported microglial clearance impairment as a key mechanistic link between B. fragilis colonization and Aβ accumulation. Abbreviations: GF: Germ-free; SPF: Specific pathogen-free; APP/PS1: Amyloid precursor protein/presenilin 1; SCFAs: Short-chain fatty acids; TMAO: Trimethylamine-N-oxide.

The gut microbiota significantly influences brain function through various signaling pathways, including: (1) the neural pathway mediated by the vagus nerve; (2) the endocrine pathway that regulates the secretion of neurotransmitters such as cortisol and serotonin; (3) the immune pathway that activates microglia and pro-inflammatory cytokines; (4) the metabolic pathway involving microbial metabolites that can cross the blood-brain barrier (BBB); and (5) the barrier integrity pathway affecting both intestinal epithelium and BBB function [5]. Preclinical and clinical studies provide preliminary evidence suggesting that gut dysbiosis may not only be a consequence of AD, but could also serve as a contributing pathogenic factor. In this context, investigating the interplay between gut microbiota and AD pathogenesis is essential for enhancing mechanistic understanding and identifying novel therapeutic targets. Microbiota-based interventions—such as probiotics, prebiotics, and fecal microbiota transplantation (FMT)—have shown promising cognitive benefits in AD animal models [1, 6]. Thus, elucidating the mechanisms of gut-brain communication, characterizing key metabolic pathways, and identifying actionable targets have become pivotal directions in current AD research.

## Methods

To ensure transparency and reproducibility, we conducted a structured literature search across three electronic databases: PubMed, Web of Science, and Scopus. The search encompassed publications from January 2010 to May 2025 and utilized a combination of the following keywords and MeSH terms: “Alzheimer’s disease,” “gut microbiota,” “microbial metabolites,” “short-chain fatty acids (SCFAs),” “tryptophan,” “trimethylamine-N-oxide (TMAO),” “indole derivatives,” “polyphenols,” “bile acids,” and “brain–gut axis.”

Inclusion criteria were as follows: 1) Original research articles (including animal experiments, clinical studies, and meta-analyses) reporting on gut microbial metabolites in the context of Alzheimer’s disease; 2) Studies reporting outcomes such as cognitive function, Aβ pathology, neuroinflammation, or gut–brain signaling mechanisms; 3) Articles published in English and in peer-reviewed journals.

Exclusion criteria included: 1) Non-peer-reviewed literature (e.g., conference abstracts and preprints); 2) Editorial opinions or commentaries; 3) Non-systematic narrative reviews, except those providing unique mechanistic insights.

The search initially retrieved 278 records. After screening titles and abstracts, 122 studies were selected for full-text review. Among these, 92 studies met all inclusion criteria and were included in the final synthesis. Representative studies were chosen based on scientific rigor, clarity of metabolite–AD associations, and mechanistic relevance, as summarized in Table 1. To contextualize the strength of the included evidence, we employed the following hierarchy of study designs: human randomized controlled trials (RCTs) > cohort studies > animal experiments. Although several clinical studies and meta-analyses were identified, the majority of included research exhibited heterogeneous designs, varied outcome measures, and inconsistent reporting formats, which limited the feasibility of meaningful quantitative synthesis. Consequently, a formal meta-analysis was not conducted, and the present review remains narrative in nature.

### Clinical and neuropathological features of Alzheimer’s disease

AD is a chronic, progressive neurodegenerative disorder primarily characterized by memory impairment as an initial symptom. Clinically, it progresses from mild cognitive decline to severe loss of daily functioning, ultimately leading to global dementia. According to the diagnostic framework established by the National Institute on Aging and the Alzheimer’s Association (NIA-AA), AD is classified into three stages: preclinical AD, mild cognitive impairment due to AD (MCI), and dementia due to AD [7].

In the early stage, patients commonly exhibit recent memory loss, attention deficits, mild language impairment, and visuospatial disorientation. As the disease advances, emotional instability, personality changes, executive dysfunction, and neuropsychiatric symptoms, such as delusions, hallucinations, and aggression, may arise. In the terminal stage, patients typically lose the ability to live independently and become entirely reliant on caregivers.

Neuropathologically, the defining lesions of AD include the extracellular deposition of β-amyloid (Aβ) peptides, which form senile plaques, and the intracellular accumulation of hyperphosphorylated tau protein, which leads to neurofibrillary tangles (NFTs) [8, 9]. Aβ is generated through the sequential cleavage of amyloid precursor protein (APP) by β- and γ-secretases, with excessive aggregation believed to induce neurotoxicity, immune activation, synaptic dysfunction, and neuronal loss [9, 10]. NFTs, composed of abnormally phosphorylated tau, disrupt microtubule stability and axonal transport, with their spatial distribution strongly correlating with cognitive decline [8, 11]. Additionally, AD pathology is characterized by widespread neuronal loss, reduced synaptic density, microglial activation, and astrogliosis in affected brain regions [8, 12].

Neuroimaging studies have confirmed the characteristic progression of structural and functional brain changes in AD. Initially, atrophy is localized to the medial temporal lobe, particularly the hippocampus and parahippocampal gyrus, which are critically involved in memory consolidation [13, 14]. As AD progresses, cortical atrophy extends to the parietal, frontal, and posterior cingulate cortices. Functional magnetic resonance imaging (fMRI) and positron emission tomography (PET) studies have revealed hypometabolism and disrupted functional connectivity in these regions, indicating pathological remodeling at the network level [14, 15]. Furthermore, PET imaging utilizing Aβ- or tau-specific tracers facilitates *in vivo* visualization of AD pathology, thereby enhancing early diagnosis and disease monitoring [16].

Multiple risk factors have been associated with AD, including advanced age, genetic predisposition, such as the presence of the apolipoprotein E ɛ4 (ApoE ɛ4) allele, chronic metabolic conditions (e.g., diabetes and hypertension), detrimental lifestyle behaviors (e.g., physical inactivity and poor diet), and psychosocial factors (e.g., depression, social isolation) [17, 18]. Familial AD is uncommon and typically linked to early-onset cases caused by mutations in *PSEN1*, *PSEN2*, or *APP*. In contrast, sporadic AD constitutes the majority of cases and involves a multifactorial etiology that includes oxidative stress, mitochondrial dysfunction, calcium dysregulation, and chronic neuroinflammation [19].

Notably, neuroinflammation has emerged as a central pathological mechanism in AD. Microglia, the primary immune effector cells in the brain, become activated in response to Aβ deposition and release pro-inflammatory cytokines such as interleukin-1β (IL-1β) and tumor necrosis factor-alpha (TNF-α), which exacerbate neurotoxicity [12, 20].

In addition to genetic and lifestyle-related risk factors, increasing attention has been directed toward the gut microbiome as a modifiable factor in AD. This has led to a growing interest in the microbial metabolites produced by the gut microbiota and their potential roles in disease progression. Accumulating evidence suggests that gut microbiota dysbiosis may influence central immune and inflammatory responses via the gut–brain axis, potentially contributing to the pathology of early-stage AD.

### Overview of the biosynthesis and functional characteristics of gut microbial metabolites

The human gastrointestinal tract contains over 10^1^^4^ microorganisms, creating a highly diverse and complex ecological system. Through the fermentation of dietary components, mucin glycoproteins, and host secretions, gut microbiota produce a wide variety of low-molecular-weight metabolites [21]. These metabolites not only support local intestinal homeostasis but can also traverse the intestinal barrier to affect distant organs, including the central nervous system, through neural, endocrine, and immune pathways [22, 23]. Notable metabolites include short-chain fatty acids (SCFAs), tryptophan-derived metabolites, secondary bile acids, amine derivatives such as trimethylamine N-oxide (TMAO), and polyphenol-derived compounds. These molecules perform various biological functions, including signal transduction, energy regulation, immune modulation, and barrier maintenance. SCFAs, primarily acetate, propionate, and butyrate, are the principal fermentation products of indigestible carbohydrates by gut microbes [21].

The production of SCFAs is contingent upon specific bacterial taxa, including *Faecalibacterium prausnitzii*, *Roseburia* spp., and *Akkermansia muciniphila*. SCFAs serve as vital energy sources for colonocytes and play a crucial role in regulating host immune responses, inflammatory signaling, and neurotransmitter synthesis. This regulation occurs through the activation of G protein-coupled receptors, such as GPR41 and GPR43, or through the inhibition of histone deacetylases (HDACs) [21, 24]. Among these SCFAs, butyrate is particularly recognized for its ability to enhance intestinal barrier integrity, promote the differentiation of regulatory T cells, and suppress pro-inflammatory cytokines. Its neuroprotective potential has also garnered significant attention [21].

Tryptophan, an essential amino acid, is metabolized by both host and microbial enzymes into various bioactive compounds. Specifically, gut bacteria convert tryptophan into indole and its derivatives, including indole-3-acetic acid (IAA), indole-3-propionic acid (IPA), and indolelactic acid (ILA). These metabolites can activate the aryl hydrocarbon receptor (AhR) and pregnane X receptor (PXR), thereby modulating inflammation, oxidative stress, and intestinal epithelial barrier function [23, 25]. Notably, some indole derivatives are capable of crossing the blood-brain barrier (BBB), influencing neuronal excitability and glial cell activation, which underscores their significant role in the modulation of brain function [23, 26].

TMAO is produced through the microbial metabolism of dietary choline, L-carnitine, and porphyrin compounds. Its precursor, trimethylamine (TMA), undergoes oxidation to form TMAO via hepatic flavin-containing monooxygenase 3 (FMO3). Elevated levels of TMAO have been positively linked to various chronic diseases, including cardiovascular disease, renal dysfunction, and neurodegenerative disorders. These associations are believed to involve mechanisms such as endothelial dysfunction, oxidative stress, and the activation of pro-inflammatory signaling pathways [27, 28]. In the context of AD, increased TMAO levels have been associated with cognitive decline and Aβ deposition. Additionally, the gut microbiota can metabolize dietary polyphenols into bioactive compounds, such as protocatechuic acid and phenylpropanoid derivatives. These metabolites play a role in free radical scavenging, modulation of low-grade inflammation, and neuroprotection [29].

Microbial-derived secondary bile acids play a significant role in regulating host metabolism and immune responses through receptors such as the farnesoid X receptor (FXR) and Takeda G protein–coupled receptor 5 (TGR5) [23]. The biosynthetic profile of these metabolites is shaped by host dietary habits, microbial composition, and the intestinal metabolic microenvironment. Their biological effects are influenced by molecular structure, concentration, receptor-binding capacity, and exhibit regional variations (e.g., between the colon and small intestine) as well as inter-individual differences. Recent advancements in metabolomics and multi-omics approaches have enabled a systematic analysis of these metabolic pathways, providing a theoretical framework for understanding the microbiota–gut–brain communication network.

### The microbiota–gut–brain axis: A bidirectional signaling network linking the gut microbiota to the brain

The microbiota–gut–brain (MGB) axis represents a complex, bidirectional communication system integrating neural, endocrine, immune, and metabolic networks that uphold homeostatic balance between the gut microbiota and the central nervous system (CNS). This system not only oversees gastrointestinal function but also significantly influences cognition, mood, stress responses, and neurodegeneration. Emerging evidence indicates that the gut microbiota impacts the CNS through various interconnected pathways, encompassing neural, endocrine, immune, metabolic, and barrier-associated mechanisms.

### Neural pathway

The neural pathway serves as the primary channel for gut-brain communication, primarily involving the vagus nerve and the enteric nervous system (ENS). The vagus nerve operates as a bidirectional conduit between the gut and the brain; its sensory terminals are located within the intestinal epithelium and muscularis, enabling the detection of microbial metabolites such as SCFAs and neuroactive compounds. These signals are transmitted to key brain regions, including the nucleus tractus solitarius, hypothalamus, and amygdala [30, 31].

The ENS can autonomously monitor luminal conditions and regulate gastrointestinal motility and secretion through local reflex arcs. Certain gut bacteria synthesize neurotransmitters or their precursors, including γ-aminobutyric acid (GABA), serotonin (5-HT), and dopamine, thereby influencing central neurotransmitter levels [32]. Notably, GABA interacts with GABA_B receptors at vagal nerve terminals, inhibiting excitatory neuronal activity in the central nervous system (CNS) and creating a negative feedback loop [31].

Although serotonin and dopamine are unable to cross the blood–brain barrier, their microbial precursors may indirectly affect central nervous function by activating vagal afferents or modulating the supply of precursors to the brain.

### Endocrine pathway

The gut functions as one of the largest endocrine organs, containing a significant number of enteroendocrine cells (EECs) that secrete hormones in response to microbial metabolites. SCFAs can activate GPR41 and GPR43 receptors on EECs, leading to the release of glucagon-like peptide-1 (GLP-1) and peptide YY (PYY). This process plays a crucial role in modulating appetite, glucose metabolism, and neuroprotection [33–35].

SCFAs activate GPR41 and GPR43 receptors on EECs, stimulating the release of GLP-1 and PYY. This process modulates appetite, glucose metabolism, and neuroprotection. These hormones exert their effects by interacting with GLP-1 receptors in the pancreas and brain, enhancing insulin secretion, regulating hypothalamic activity, and promoting neuronal survival. Tryptophan-derived metabolites also influence the synthesis of melatonin and serotonin through endocrine pathways. Although these hormones do not cross the blood-brain barrier directly, they can affect brain function by acting on peripheral receptors and modulating vagal afferents and hypothalamic pathways [36]. Specifically, GLP-1 and PYY influence hypothalamic-pituitary-adrenal (HPA) axis activity by regulating corticotropin-releasing hormone (CRH) expression in the hypothalamus and subsequent cortisol secretion, thereby affecting stress responses and neuroinflammation. Additionally, the gut microbiota modulates HPA axis responsiveness, impacting stress-related hormones such as cortisol, as well as central inflammation and synaptic plasticity.

### Immune pathway

The gut microbiota plays a crucial role in the development and functioning of the host immune system. It regulates the differentiation of dendritic cells, macrophages, and regulatory T cells (Tregs), thereby influencing both local and systemic immune responses. Short-chain fatty acids (SCFAs), particularly butyrate, promote Treg differentiation, inhibit pro-inflammatory Th17 cell activity, and reduce the production of cytokines such as IL-6, IL-1β, and TNF-α [37, 38]. Activated immune cells and circulating cytokines can affect neuroinflammatory states and activate microglia. Specifically, pro-inflammatory mediators such as interleukin-1β (IL-1β) and tumor necrosis factor-alpha (TNF-α) can trigger microglial activation via NF-κB signaling, leading to increased Aβ accumulation and neuronal damage. Indole derivatives modulate immune responses and intestinal barrier integrity through the aryl hydrocarbon receptor (AhR) signaling pathway, playing a vital role in the immuno-neural axis [39]. Upon activation by indole compounds, AhR translocates to the nucleus and regulates the expression of interleukin-22 (IL-22), which promotes mucosal healing and enhances the expression of epithelial tight junction proteins, thereby reinforcing gut barrier function and mitigating systemic inflammation [40]. Furthermore, dysbiosis-induced translocation of microbial endotoxins, such as lipopolysaccharide (LPS), may increase systemic inflammation and BBB permeability, exacerbating CNS pathology [2, 41].

### Metabolic pathway

Microbial metabolites—including SCFAs, tryptophan-derived compounds, secondary bile acids, TMAO, and polyphenol derivatives—play a crucial role in mediating host-microbiota interactions. Certain small metabolites, such as acetate and propionate, can traverse the BBB via systemic circulation, thereby directly influencing neuronal excitability and synaptic function [42]. SCFAs have been shown to regulate neurotransmitter synthesis, neurogenesis, and the immune states of the central nervous system (CNS) through G protein-coupled receptor (GPR) signaling and histone deacetylase (HDAC) inhibition [43]. Specifically, the activation of GPR41 and GPR43 by SCFAs modulates microglial activity and promotes the production of anti-inflammatory cytokines. Furthermore, HDAC inhibition by butyrate enhances the expression of brain-derived neurotrophic factor (BDNF), which supports neuronal differentiation [44, 45]. TMAO, a nitrogenous metabolite, is linked to cognitive impairment, Aβ deposition, and neuroinflammation, potentially through mechanisms involving oxidative stress, endothelial dysfunction, and the upregulation of inflammatory mediators [42, 46]. Additionally, bile acid derivatives and microbial-converted polyphenols can influence neuronal energy metabolism and mitochondrial function, thereby contributing to the progression of neurodegenerative diseases [47]. For example, secondary bile acids such as lithocholic acid (LCA) activate TGR5 receptors in neuronal and glial cells, promoting mitochondrial biogenesis and mitigating oxidative stress. Similarly, microbial metabolites of dietary polyphenols, such as urolithin A, enhance mitophagy and ATP production in neurons, thus providing neuroprotective effects [48, 49].

### Barrier interaction pathway

This pathway highlights the collaborative function of the intestinal epithelial barrier and the BBB in sustaining the homeostasis of the microbiome-gut-brain (MGB) axis. An intact gut barrier, supported by tight junction proteins such as occludin and claudins, effectively prevents the translocation of pro-inflammatory molecules. However, microbial dysbiosis can compromise this barrier, allowing microbial products and cytokines to enter the bloodstream, which may result in CNS inflammation and disruption of the microenvironment. SCFAs enhance the expression of tight junction proteins, restore barrier architecture, and stabilize intestinal permeability [50]. Concurrently, the integrity of the BBB is influenced by gut-derived signals. Research involving germ-free (GF) mice has demonstrated increased BBB permeability, which can be partially restored through SCFA supplementation. Additionally, microbial metabolites may further affect BBB transport properties and the neuroimmune microenvironment by interacting with cerebral endothelial cells, basement membranes, and astrocytic end-feet [51].

Recent studies have enhanced our understanding of how gut microbiota modulates CNS glial cell function and neuroinflammation in AD. A comprehensive review by Caradonna et al. [52] highlighted the regulatory effects of gut-derived signals on microglia, astrocytes, and oligodendrocytes through SCFA and cytokine-mediated pathways. Additionally, research indicated that the conditions of animal facilities (specific pathogen-free [SPF] vs specific pathogen-free with additional environmental controls [SOPF]) significantly influence amyloid pathology in 5XFAD mice via microbiota-dependent mechanisms, emphasizing the environmental impact on gut-brain communication [53].

### Role of gut microbial metabolites in the pathogenesis of Alzheimer’s disease

AD encompasses multifactorial pathological processes, including Aβ deposition, hyperphosphorylation of tau protein, neuronal apoptosis, neuroinflammation, and synaptic dysfunction. Emerging evidence indicates that gut microbial metabolites may influence these CNS pathologies through various mechanisms and may play a role in modulating disease progression, even in the early stages of AD. Certain metabolites are capable of crossing the BBB to directly impact neurons or indirectly influence neurodegenerative progression through inflammatory signaling, oxidative stress, and synaptic plasticity [54, 55]. Notably, SCFAs, particularly butyrate, demonstrate neuroprotective potential in AD models by modulating immune responses and maintaining BBB integrity [51].

Their context-dependent effects on microglial activation and Aβ pathology highlight the significance of host status and treatment timing in therapeutic applications. Numerous studies have established the neuroprotective roles of SCFAs—particularly butyrate—in regulating inflammation, enhancing BBB integrity, and promoting synaptic plasticity; however, conflicting findings are also evident. For instance, Colombo et al. reported that SCFA supplementation in germ-free APPPS1 mice resulted in increased Aβ plaque deposition and microglial activation, indicating a potentially detrimental context-dependent effect.

**Figure 1.**
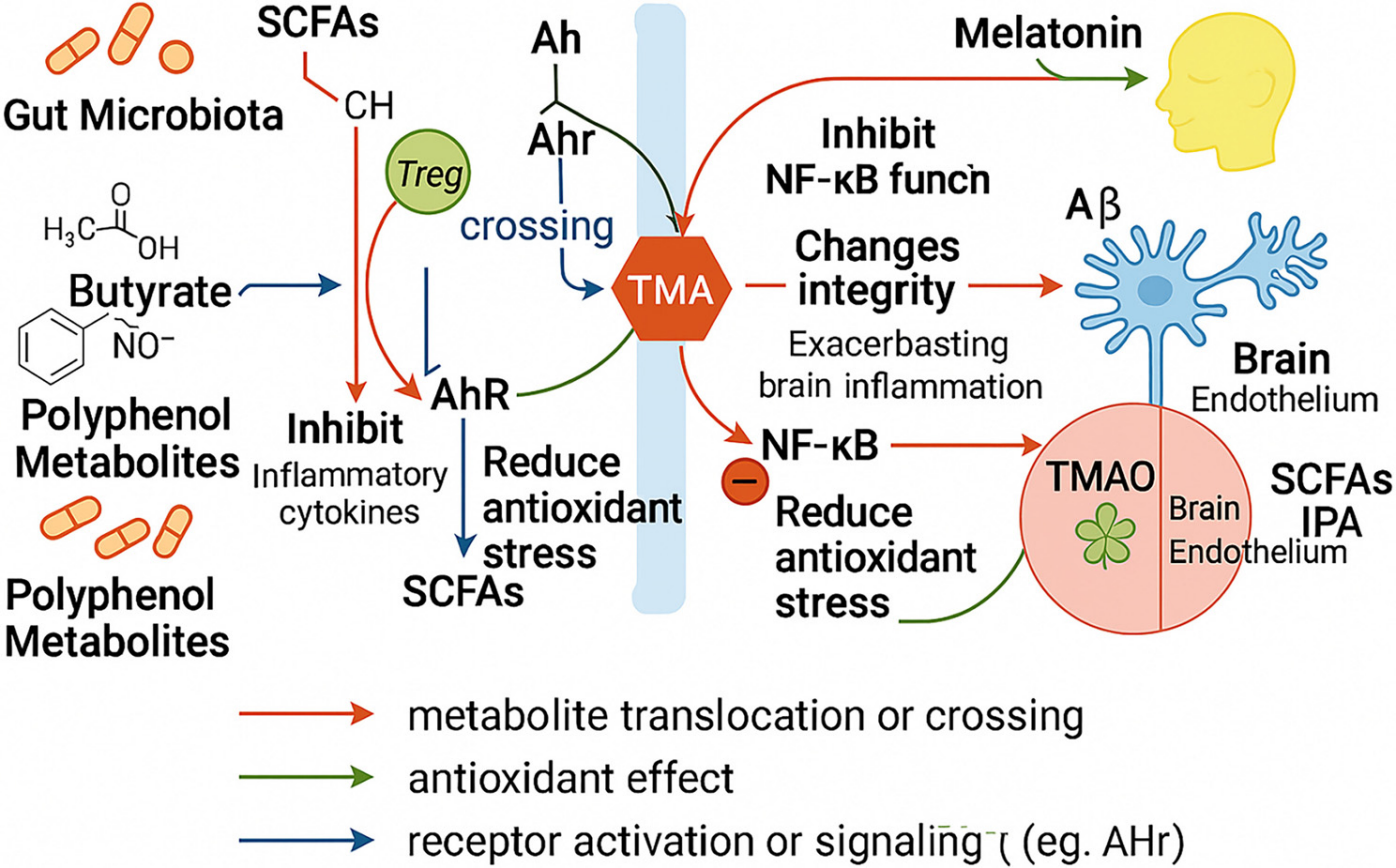
**Gut microbial metabolites regulate Alzheimer’s disease via immune, oxidative, and barrier-related mechanisms.** Gut microbiota-derived metabolites influence AD through multiple pathways. SCFAs, especially butyrate, promote Treg differentiation, inhibit pro-inflammatory cytokines, and enhance BBB integrity, but may under certain conditions increase Aβ deposition. Tryptophan metabolites (e.g., IPA) activate AhR signaling with antioxidant and anti-inflammatory effects. Polyphenol metabolites suppress NF-κB and reduce oxidative stress. In contrast, TMA and TMAO impair BBB integrity, promote Aβ accumulation, and exacerbate neuroinflammation, linking gut dysbiosis to AD pathology via the gut–brain axis. Abbreviations: AD: Alzheimer’s disease; SCFAs: Short-chain fatty acids; BBB: Blood–brain barrier; CH: Cholesterol; Treg: Regulatory T cells; AhR: Aryl hydrocarbon receptor; TMA: Trimethylamine; NF-κB: Nuclear factor kappa-light-chain-enhancer of activated B cells; Aβ: Beta-amyloid; TMAO: Trimethylamine N-oxide; IPA: Indole-3-propionic acid.

Several factors may account for these divergent outcomes. First, the germ-free status alters immune system maturation and brain development, potentially amplifying inflammatory responses to SCFA exposure. Second, the dosage and route of administration (e.g., bolus gavage vs gradual dietary intake) can differentially affect metabolic and immune responses. Third, the timing of intervention—specifically whether SCFAs are administered during early or late disease stages—may influence their impact on Aβ pathology. Consequently, SCFAs may exert bidirectional effects in AD models, contingent upon host context, microbiota status, and treatment parameters. This duality emphasizes the need for meticulous experimental design and patient stratification in translational applications.

Tryptophan-derived metabolites also serve as critical molecular mediators of the gut–brain interaction. In AD mouse models, IPA and ILA activate the AhR signaling pathway, thereby enhancing both intestinal and cerebral barrier functions while suppressing microglial pro-inflammatory phenotypic transformation. Furthermore, IPA possesses antioxidant properties, scavenging reactive oxygen species (ROS) and alleviating oxidative stress-induced neuronal injury [56, 57].

Tryptophan influences melatonin synthesis through the gut–pineal–CNS axis, which plays a crucial role in regulating circadian rhythms and providing neuroprotection. Disruptions in this process may accelerate the pathology of AD [58]. Gut-derived amine metabolites, particularly TMAO, show a positive correlation with the development of AD. Research indicates that TMAO promotes neuroinflammatory responses, upregulates Aβ precursor protein expression and cleavage, and increases Aβ accumulation [46]. Furthermore, TMAO compromises the integrity of cerebrovascular endothelial cells, leading to heightened BBB permeability and enhanced inflammatory signaling to the CNS. Elevated TMAO levels are inversely associated with cognitive function and may worsen neurodegeneration by impairing cholinergic neurotransmission, increasing oxidative stress, and disrupting mitochondrial function [59, 60].

Polyphenol derivatives and secondary bile acids play significant roles in the pathogenesis of AD. Polyphenol metabolites have been shown to inhibit the NF-κB signaling pathway, reduce the expression of proinflammatory cytokines, and enhance the activity of endogenous antioxidant enzyme systems in the brain, thereby mitigating oxidative damage [61, 62]. Certain secondary bile acids modulate neuronal energy metabolism and lipid homeostasis through the TGR5 and FXR pathways; their dysregulation is closely linked to mitochondrial dysfunction and lipid abnormalities associated with AD [63].

Multi-omics studies have identified significant changes in the gut microbial composition and metabolic profiles of AD patients. Compared to healthy controls, these individuals exhibit decreased levels of SCFAs, reduced biosynthesis of indole and polyphenol derivatives, and elevated levels of TMAO. These findings suggest that microbial metabolites may contribute to the pathogenesis of AD through multiple synergistic mechanisms [64, 65]. Integrated metabolomic and transcriptomic analyses further demonstrate that dysregulated metabolic pathways are significantly associated with inflammatory markers and Aβ-related gene expression in the brain, highlighting the central regulatory role of microbial metabolites in AD pathophysiology [66]. Collectively, these findings emphasize that gut microbial metabolites are not merely mediators of microbiota-brain communication; rather, they may act as functional effectors involved in various pathological processes of AD, including neuroinflammation, oxidative stress, neurotransmitter metabolism, and BBB dysfunction. A schematic overview of these interactions is presented in Figure 1.

### Clinical evidence of gut microbial metabolites in Alzheimer’s disease

With the advancement of the microbiota–gut–brain axis (MGB axis) theory, an increasing number of clinical translational studies have concentrated on gut microbial metabolites in AD. Numerous clinical observations, interventional trials, and metabolomic analyses have established a significant correlation between these metabolites and cognitive function, inflammatory status, and pathological protein levels in AD patients. This evidence provides a robust theoretical foundation for microbiota-targeted interventions in the context of Alzheimer’s disease.

Studies consistently demonstrate that levels of SCFAs are significantly reduced in both peripheral blood and fecal samples of patients with AD. In contrast, concentrations of TMAO are elevated, while levels of indole and its derivatives are diminished [67]. These metabolic alterations are significantly correlated with Mini-Mental State Examination (MMSE) scores, brain atrophy, and Aβ_4__2_ levels. For instance, a case-cohort analysis from the AgeCoDe study in Germany, which included 805 older adults without dementia at baseline, found that no inflammatory markers effectively predicted the onset of dementia. However, certain indole-containing tryptophan metabolites and SCFAs—specifically isobutyric acid and 2-methylbutyric acid—were significantly associated with the progression to all-cause dementia, although SCFAs and TMAO were not independently predictive in adjusted models [68].

Several interventional studies have investigated the functional roles of microbial metabolites in microbiota-based therapies, including probiotic supplementation and fecal microbiota transplantation (FMT). A double-blind randomized controlled trial conducted in Italy reported that a combined probiotic intervention—featuring *Lactobacillus* and *Bifidobacterium* strains—significantly increased peripheral SCFA concentrations in patients with AD, which was accompanied by improved MMSE scores after 12 weeks [69, 70]. Preliminary findings from a 2023 study in China further demonstrated that FMT could remodel gut microbial composition, restore SCFA and indole metabolic pathways, reduce inflammatory markers, and indicate a trend toward cognitive improvement [71]. Additionally, metabolomic studies integrated with neuroimaging are advancing. A 2022 study in the United States employed ultra-high-performance liquid chromatography-tandem mass spectrometry (UHPLC–MS/MS) to quantify metabolomic profiles and utilized positron emission tomography (PET) imaging to assess Aβ burden. The results indicated that elevated plasma levels of indole-3-propionic acid (IPA) were associated with reduced Aβ deposition, suggesting a neuroprotective role for this metabolite in regulating Aβ pathology [72].

Numerous translational experiments have sought to modulate microbial metabolic pathways through nutritional and pharmacological interventions. Supplementation with resistant starch, low-protein diets, and oral sodium butyrate has demonstrated efficacy in enhancing cognitive function and reducing neuroinflammation in animal models of AD. Additionally, these interventions have been associated with increased SCFA levels in healthy elderly individuals, providing preliminary evidence for the clinical feasibility of such approaches [2, 42, 73]. Table 1 presents a summary of key published studies, outlining changes in critical microbial metabolites—such as SCFAs and TMAO—in AD populations, along with relevant intervention strategies and their potential therapeutic effects. The significant findings on gut microbial metabolites in Alzheimer’s disease are detailed in Table 1.

### Potential therapeutic targets of gut-derived metabolites in Alzheimer’s disease

In recent years, the modulation of gut microbiota has emerged as a promising therapeutic strategy for AD. Targeting microbial metabolites offers a direct intervention at the microbe-brain interface. Various metabolites derived from gut microbiota have shown regulatory effects on critical AD-related pathological processes, including neuroinflammation, oxidative stress, and Aβ metabolism. These metabolites may represent novel therapeutic targets for personalized interventions. The subsequent section delineates the therapeutic mechanisms and recent advancements in several key categories of microbial metabolites.

### Short-chain fatty acids (SCFAs)

SCFAs, particularly butyrate, have demonstrated therapeutic potential in AD by modulating inflammation, oxidative stress, and the integrity of the BBB. Butyrate promotes an anti-inflammatory microglial phenotype and enhances synaptic plasticity. Interventions such as oral sodium butyrate and probiotics that increase SCFA production are currently being investigated for their neuroprotective effects. Furthermore, butyrate facilitates the polarization of microglia toward the anti-inflammatory M2 phenotype while inhibiting the pro-inflammatory M1 activation commonly associated with AD [77]. The oral administration of sodium butyrate and butyrate-producing probiotics is being explored as a potential therapeutic strategy [2].

### Tryptophan-derived metabolites

Tryptophan metabolism plays a crucial role in gut-brain communication and occurs through three primary pathways: the kynurenine (KYN) pathway, serotonin synthesis, and microbial indole production. The KYN pathway and its metabolites are significant regulators of CNS inflammation and neurotoxicity, particularly in AD [78]. In AD, tryptophan is predominantly metabolized into neurotoxic KYN derivatives, such as 3-hydroxykynurenine, due to the upregulation of indoleamine 2,3-dioxygenase (IDO), which contributes to neuronal damage and microglial activation [79]. Concurrently, levels of neuroprotective metabolites like kynurenic acid (KYNA) decline, thereby disrupting metabolic homeostasis [80, 81]. Consequently, therapeutic strategies are focused on redirecting tryptophan metabolism toward protective pathways while suppressing the production of neurotoxic byproducts.

### Indole derivatives

IPA is a gut microbial metabolite synthesized from tryptophan, predominantly by bacterial genera such as Clostridium and Bacteroides. In addition to its role in amyloid regulation, IPA modulates glial cell activity and suppresses neuroinflammatory gene expression in the hippocampus. Importantly, IPA can cross the BBB, inhibit Aβ aggregation, and prevent neuronal apoptosis, thereby exhibiting significant neuroprotective effects [61]. Strategies to enhance the production of indole derivatives—either through the supplementation of indole-producing bacteria or the administration of synthetic metabolites—have shown cognitive benefits and a reduction in pathology in AD models [56].

### TMAO and other amine metabolites

Given its pathogenic role in AD, therapeutic strategies targeting the production of TMAO have emerged. These strategies include dietary restrictions on precursors such as choline and L-carnitine, as well as the use of microbial enzyme inhibitors. The objective of these approaches is to mitigate TMAO-induced neuroinflammation and disruption of the BBB.

A recent review by Oktaviono et al. [82]. published in *Biomolecules and Biomedicine* elaborates on the pathophysiological roles of TMAO and discusses potential therapeutic modulation strategies within the context of AD, providing valuable mechanistic insights that complement our discussion. Concurrently, recent mechanistic and epidemiological studies have reinforced the detrimental role of TMAO in cognitive decline. A meta-analysis involving over 80,000 participants confirmed that elevated plasma TMAO is significantly associated with cognitive impairment, indicating an odds ratio of approximately 1.39 [59]. *In vivo* studies have demonstrated that TMAO induces neuronal senescence, exacerbates oxidative stress, disrupts mTOR signaling, and exacerbates neuroinflammation [27]. Furthermore, comprehensive reviews published between 2023 and 2024 have summarized TMAO’s negative impacts on endothelial and synaptic function and suggested potential therapeutic strategies that target dietary precursors and microbial enzymes [83].

### Other gut-derived metabolites

Beyond the previously mentioned metabolites, secondary bile acids, such as deoxycholic acid and lithocholic acid, exert regulatory effects in AD by modulating energy metabolism, lipid homeostasis, and neurodevelopment through receptors including TGR5 and FXR [84, 85]. Polyphenol-derived microbial metabolites have exhibited antioxidant, anti-inflammatory, and neuroprotective properties, potentially delaying AD progression by optimizing metabolic interactions [86, 87]. Emerging candidates, such as GABA, N-acetylneuraminic acid, and short-chain hydroxy fatty acids, are also gaining prominence [88]. Although these molecules are synthesized endogenously in the CNS, their peripheral microbial origins and modulatory potential are increasingly being investigated. Notably, elevated GABA levels have been linked to improved cognition in AD models, although the relationship between gut-derived GABA and central GABAergic signaling requires further elucidation [88]. In summary, gut-derived metabolites play a crucial role as effector molecules in the pathogenesis of AD and represent promising targets for precision therapeutic interventions.

### Limitations and future directions

Emerging evidence supports the involvement of gut microbiota–derived metabolites in the pathogenesis and intervention of AD; however, several critical challenges must be addressed. First, most current studies rely on animal models or small-sample clinical trials, and their translational applicability and safety in large, diverse human populations have yet to be rigorously validated [89]. Second, the brain–gut–microbiota axis constitutes a complex, multilayered communication network that encompasses neural, endocrine, immune, and metabolic pathways. Nevertheless, mechanistic insights into key signaling molecules and their spatiotemporal dynamics remain limited, particularly concerning differential responses across disease stages and individual variability. Furthermore, the levels of microbial metabolites are significantly influenced by extrinsic factors such as diet, age, and lifestyle, and there is currently no unified or standardized protocol for their detection, quantification, or clinical interpretation. This lack of standardization impedes the utilization of gut-derived metabolites as reliable non-invasive biomarkers in clinical settings.

Future research should incorporate multi-omics approaches—including metabolomics, metagenomics, and transcriptomics—to develop multi-pathway synergy models and clarify the dynamic interactions of metabolites in relation to disease progression. Longitudinal cohort studies are crucial for monitoring temporal fluctuations in metabolite profiles and identifying disease-specific signatures across various clinical and environmental contexts. Furthermore, precision microbiome-based interventions—such as targeted prebiotics, microbial modulation, fecal microbiota transplantation (FMT), and synthetic biological therapeutics—should be assessed within individualized evaluation frameworks. These strategies offer significant potential for early intervention and long-term management of AD, ultimately facilitating the advancement of personalized gut-brain therapeutics.

## Conclusion

The role of gut microbiota–derived metabolites in Alzheimer’s disease (AD) has gradually shifted from associative comorbidity observations to mechanistic investigations, revealing their potential as therapeutic targets. Metabolites such as short-chain fatty acids (SCFAs), tryptophan derivatives, indole compounds, and trimethylamine N-oxide (TMAO) are not only involved in regulating neuroinflammation, blood–brain barrier (BBB) integrity, and neurotransmitter metabolism, but also influence the onset and progression of AD through multiple brain–gut axis signaling pathways.An increasing body of clinical and translational evidence supports the utility of gut-derived metabolites as promising biomarkers and integral components of novel intervention strategies. Although challenges remain—particularly regarding the clarification of underlying mechanisms and the standardization of clinical applications—current findings have opened new avenues for early detection and precision treatment of AD. Furthermore, this line of research provides a robust theoretical and experimental foundation for optimizing future microbiome-based therapeutic approaches.
